# Mechanical Injury Enhances Ethylene-Associated Transcriptional and Metabolic Responses of Strawberry Fruit During Shelf-Life Period

**DOI:** 10.3390/foods15193380

**Published:** 2026-09-23

**Authors:** Jiayi Liu, Haoyang Liu, Shuhan Yang, Cheng Wang, Yicheng Ren, Dong Li, Chuyan Wang, Yining Xia, Zisheng Luo, Yanpei Chen

**Affiliations:** 1 College of Biosystems Engineering and Food Science, Zhejiang University, Hangzhou 310058, China; 22413111@zju.edu.cn (J.L.); 3220104771@zju.edu.cn (H.L.); 22413043@zju.edu.cn (S.Y.); 12513072@zju.edu.cn (C.W.); renyicheng@zju.edu.cn (Y.R.); dong_li@zju.edu.cn (D.L.); luozisheng@zju.edu.cn (Z.L.); 2 Key Laboratory of Agro-Products Postharvest Handling of Ministry of Agriculture and Rural Affairs, Zhejiang Key Laboratory for Agri-Food Resources and High-Value Utilization, Zhejiang University, Hangzhou 310058, China; 3 Innovation Center for Postharvest Agro-Products Technology, Zhejiang University, Hangzhou 310058, China; 4 College of Biology, Food & Environment, Hefei University, Hefei 230601, China; wangchuyan19@163.com; 5 Institute of Food Science and Technology, Chinese Academy of Agricultural Sciences, Beijing 100193, China; xiayining@caas.cn

**Keywords:** strawberry, mechanical injury, ethylene, wound response, oxidative stress, transcriptomics, metabolomics

## Abstract

Strawberry (*Fragaria* × *ananassa*) is a typical non-climacteric fruit in which the contribution of ethylene to postharvest ripening remains controversial. Mechanical injury can induce endogenous wound ethylene production, providing an opportunity to strengthen ethylene-associated responses when combined with exogenous ethylene exposure. In this study, ‘Benihoppe’ strawberry fruit were subjected to control (CK), mechanical injury (MI), ethylene treatment (ET), and combined mechanical injury and ethylene treatment (ME). Changes in postharvest quality, ethylene biosynthesis, oxidative damage, metabolomic profiles, and transcriptomic profiles were evaluated during the shelf-life period. ME-treated fruit showed the most severe postharvest deterioration, with accelerated softening, visual quality loss, and enhanced total anthocyanin accumulation. ME treatment was associated with the highest 1-aminocyclopropane-1-carboxylic acid (ACC) accumulation, which reached 5.9 nmol g^−1^ fresh weight (FW) at day 3 and remained at 4.2 nmol g^−1^ FW at day 6, indicating strengthened ethylene biosynthesis under combined treatment. ME fruit also showed the highest relative electrolyte leakage, malondialdehyde (MDA) content, and H_2_O_2_ accumulation, indicating more severe oxidative membrane damage. Multi-omics analyses revealed extensive metabolic and transcriptional reprogramming. Comparisons of CK vs. ME, MI vs. ME, and ET vs. ME identified 4012, 2825, and 7042 differentially expressed genes, indicating that ME induced a transcriptional state distinct from both untreated and single-treatment fruit at day 6. Differentially accumulated metabolites and differentially expressed genes were mainly associated with phenylpropanoid and anthocyanin biosynthesis, sugar-acid metabolism, oxidative stress, and hormone-related transcriptional regulation. Collectively, combined mechanical injury and ethylene exposure were associated with more rapid postharvest deterioration, aggravated oxidative membrane damage, and coordinated transcriptional and metabolic remodeling in strawberry fruit.

## 1. Introduction

Strawberry (*Fragaria* × *ananassa* Duch.) is an economically important berry crop appreciated for its attractive appearance, characteristic aroma, desirable flavor, and high levels of compounds beneficial for health, including vitamin C, phenolics, flavonoids, and anthocyanins [1]. However, strawberry fruit are highly perishable after harvest because their soft texture, thin epidermis, high moisture content, and active metabolism make them sensitive to physical stress [2]. As a result, even mild collisions or compression can cause visible mechanical injuries, thereby compromising tissue integrity, accelerating water loss, promoting softening, and ultimately shortening shelf life [3]. Mechanical injury also increases the risk of microbial decay by impairing surface barriers and weakening fruit resistance to pathogen invasion [4]. At the physiological level, mechanical injury disrupts membrane integrity and cellular compartmentalization, promoting reactive oxygen species accumulation, lipid peroxidation, and other senescence-related stress responses [5]. These injury-induced responses may further disturb fruit respiration, secondary metabolism, and hormonal regulation, thereby amplifying postharvest quality loss [6]. Therefore, mechanical injury represents both a visible quality defect and a postharvest stress signal that limits the quality, safety, and commercial value of strawberry fruit.

Fleshy fruit are commonly classified as climacteric or non-climacteric based on respiratory behavior and ethylene dependence during ripening. In climacteric fruit, such as tomato and banana, ripening involves a sharp increase in respiration together with elevated ethylene production, while being largely regulated by ethylene [7]. In contrast, non-climacteric fruit generally produce low levels of ethylene and show limited phenotypic responses to exogenous ethylene. Strawberry is typically regarded as a non-climacteric fruit, with ripening primarily associated with abscisic acid (ABA). Nevertheless, recent evidence indicates that ethylene also contributes to strawberry ripening and senescence, although its role appears to be more dependent on developmental stage and coordination with other hormonal pathways than that of a dominant ripening trigger [8]. Genes related to ethylene biosynthesis, perception, and signaling exhibit development-dependent expression patterns in strawberry, suggesting that ethylene may contribute to ripening in a tissue-specific or stage-dependent manner [9,10,11]. Ethylene signaling components such as *FaCTR1* are associated with strawberry ripening regulation, providing genetic evidence for ethylene involvement in this process [12]. In addition, strawberry fruit red coloration can be slightly accelerated by ethylene or inhibited by 1-methylcyclopropene (1-MCP) [13,14]. Ethylene treatment can also influence cell wall metabolism, including changes in hemicellulose, cellulose, neutral sugars, and cell wall-modifying enzymes [15]. Ethylene elicitors or inhibitors can also alter strawberry fruit development and metabolism, with effects depending on the developmental stage of the fruit [16]. Moreover, ethylene was shown to promote ABA biosynthesis in postharvest strawberry fruit through the miR161-*FaNCED1* module, further supporting ethylene–ABA crosstalk during postharvest ripening and senescence [17]. Together, these findings indicate that ethylene is not the dominant ripening regulator in strawberry, but it may still participate in ripening and senescence in a stage-dependent and condition-dependent manner. However, the relatively low endogenous ethylene production of strawberry fruit makes it difficult to distinguish direct ethylene effects from broader developmental and stress-related processes. Therefore, experimental approaches that enhance ethylene biosynthesis and ethylene-associated responses may provide a useful strategy for clarifying the role of ethylene in postharvest metabolic regulation.

During commercial distribution and storage, strawberry fruit may experience mechanical stress and simultaneous exposure to low levels of ethylene that accumulate in closed packaging environments. However, most studies have examined mechanical injury or ethylene exposure separately, leaving the integrated physiological, metabolic, and transcriptional responses to their combined exposure poorly understood. It has been reported that mechanical injury can induce wound ethylene production in kiwifruit and persimmon by increasing the activity or expression of key ethylene biosynthetic enzymes, including 1-aminocyclopropane-1-carboxylic acid (ACC) synthase (ACS) and ACC oxidase (ACO) [18,19]. Because mechanical injury also activates jasmonate-related and other stress responses, wound ethylene may interact with broader stress signaling networks and modify fruit responses to ethylene-containing environments [20]. Given this context, we therefore hypothesized that mechanical injury-induced wound responses would alter fruit responses to exogenous ethylene, resulting in more severe deterioration than either treatment alone. This study investigated the physiological, transcriptional, and metabolic responses of strawberry fruit to mechanical injury, ethylene exposure, and their combination.

## 2. Materials and Methods

### 2.1. Plant Material and Treatments

Strawberry fruit (*Fragaria* × *ananassa* Duch. cv. ‘Benihoppe’) were harvested at the white stage from a commercial orchard in Hangzhou, China (approximately 30.332° N, 120.211° E). At this developmental stage, the fruit had reached nearly full size but had not yet developed visible red coloration. The white stage precedes the rapid coloration and softening associated with strawberry ripening and is therefore frequently selected as a starting point for monitoring ripening-related physiological and quality changes [8,21]. Fruit with uniform size and maturity without visible disease, defects, or mechanical damage were selected. The fruit were transported to the laboratory in insulated boxes containing ice packs and sorted within 2 h of harvest.

Fruit were randomly divided into four groups: CK, MI, ET, and ME. CK fruit received neither mechanical injury nor ethylene treatment. For MI treatment, ten puncture wounds were made evenly around the equatorial region of each fruit using a sterile dissecting needle, with each wound approximately 3 mm deep. For ET treatment, two commercial controlled-release ethephon-based ethylene agent sachets (Dalian Xinyang Import and Export Co., Ltd., Dalian, China) were used in each package. The sachets gradually released ethylene through the decomposition of ethephon after activation. Before use, the sachets were activated in a 50 °C water bath for 5 min according to the manufacturer’s instructions. The ethylene concentration in the package headspace was approximately 160–200 ppm throughout the experiment. ME fruit were first subjected to the same mechanical injury treatment as MI fruit and were then exposed to the activated ethylene-releasing sachets following the same procedure as ET fruit.

Fruit from all four treatment groups were placed in the same type of polyethylene bags (commercially available conventional polyethylene bags with no claimed specialized functional properties), which were sealed and maintained under identical packaging, handling, and shelf-life conditions. All fruit were kept at 20 ± 1 °C and 85–90% relative humidity to establish an accelerated postharvest deterioration model. Each treatment contained 18 fruit and consisted of three independent biological replicates. Samples were collected after 0, 3, and 6 d of the shelf-life period. Technical measurements were performed in triplicate when applicable and averaged before statistical analysis. Samples used for transcriptomic, metabolomic, and RT-qPCR analyses were immediately frozen in liquid nitrogen and stored at −80 °C until analysis.

### 2.2. Quality Attributes

Fruit appearance was recorded using a digital camera (SONY A7M2, Sony Corporation, Tokyo, Japan). Firmness was measured according to Chen [22] with modifications, using a texture analyzer (TA-XT Plus, Stable Micro Systems Ltd., Godalming, Surrey, UK) equipped with a P/2 cylindrical probe. The P/2 probe has a diameter of 2 mm and a contact area of approximately 3.14 mm^2^. After removing the peel at the equatorial region, each fruit was punctured three times at a speed of 1 mm s^−1^ to a depth of 5 mm. The maximum penetration force was expressed as firmness in Newtons (N).

Weight loss was calculated as:

(1) Weight loss (%) = (*W*_0_ − *W_t_*)/*W*_0_ × 100

where *W*_0_ is the initial fruit weight and *W_t_* is the fruit weight at each sampling time. Soluble solids content was measured using a digital refractometer and expressed as °Brix. Titratable acidity was determined by acid–base titration and expressed as citric acid equivalent. Total anthocyanin content was measured using an anthocyanin assay kit (G0126F, 48 samples, Suzhou Comin Biotechnology Co., Ltd., Suzhou, China) according to the manufacturer’s instructions. Total anthocyanin content was expressed as mg 100 g^−1^ FW.

### 2.3. Ethylene Production and Ethylene Biosynthesis-Related Parameters

Ethylene production was determined following the method described by Yang [23]. Fruit were sealed in airtight containers of 2 L for 1 h. Then, 1 mL of headspace gas was withdrawn and injected into a gas chromatograph equipped with a GDX-502 column. The injector, column, and detector temperatures were 120, 80, and 150 °C, respectively. Ethylene concentration was calculated using a standard curve prepared with standard ethylene gas and expressed as μL kg^−1^ h^−1^ on a fresh weight basis. ACC content was determined according to Tong [24] with modifications. Frozen samples were ground under liquid nitrogen, and 50 mg of powder was extracted with 1.0 mL of pre-cooled 80% ethanol. The mixture was vortexed for 2 min, ultrasonicated in an ice bath for 10 min, and centrifuged at 15,000× *g* for 10 min at 4 °C. Approximately 800 μL of the supernatant was filtered through a 0.22-μm hydrophilic polytetrafluoroethylene (PTFE) membrane and analyzed by UPLC-qTOF-MS/MS in positive-ion mode using an ACQUITY UPLC HSS T3 column (100 × 2.1 mm, 1.8 μm) at 35 °C. ACC was quantified against standards prepared in 80% ethanol (0.5–500 ng mL^−1^) and expressed as nmol g^−1^ FW.

### 2.4. Oxidative Damage and Antioxidant Enzyme Activities

Relative electrolyte leakage was determined according to Dionisio-Sese [25], with minor modifications. Fruit discs were rinsed and immersed in deionized water. Initial conductivity was recorded as *C*_1_. The samples were then boiled, cooled to room temperature, and final conductivity was recorded as *C*_2_. Relative electrolyte leakage was calculated as:

(2) Relative electrolyte leakage (%) = *C*_1_/*C*_2_ × 100

Hydrogen peroxide (H_2_O_2_) content was determined using the titanium sulfate colorimetric method [26]. Malondialdehyde (MDA) content was measured using the thiobarbituric acid method [27] based on absorbance at 532, 600, and 450 nm and expressed as nmol g^−1^ FW. Peroxidase (POD) activity was measured using the guaiacol method [28]. One unit of POD activity was defined as an increase of 0.01 in absorbance at 470 nm per min. Superoxide dismutase (SOD) activity was determined using the nitroblue tetrazolium photoreduction inhibition method [29]. One unit of SOD activity was defined as the enzyme amount required to inhibit NBT photoreduction by 50%.

### 2.5. Metabolomic Analysis

Samples collected from CK, MI, ET, and ME fruit on day 6 were used for metabolomic analysis, with three biological replicates for each treatment. Day 6 was selected because treatment-dependent differences in fruit appearance and physiological and quality attributes were most pronounced at this time point, allowing metabolomic characterization of the established late-stage responses. Metabolite extraction and LC–MS analysis were performed as described by Li [30], with minor modifications. Frozen samples were ground in liquid nitrogen, and metabolites were extracted using a methanol–water system. Extracts were analyzed by UPLC-qTOF-MS/MS. Chromatographic separation was performed on an ACQUITY UPLC HSS T3 column, and mass spectrometry was conducted with an electrospray ionization source in positive and negative ion modes. Raw data were processed for peak detection, alignment, filtering, and normalization. Metabolites were annotated using Human Metabolome Database (HMDB) and Kyoto Encyclopedia of Genes and Genomes (KEGG) databases. Equal aliquots of all samples were pooled as QC samples. Quality control (QC) samples were injected before formal analysis to equilibrate the system and were inserted every 6–10 samples during acquisition to monitor analytical stability. Features with relative standard deviation (RSD) < 30% in QC samples were retained for further analysis. Differentially accumulated metabolites were screened using variable importance in projection (VIP) > 1.0 and *p* < 0.05. KEGG enrichment analysis was performed for differential metabolites.

### 2.6. Transcriptome Sequencing and Analysis

Transcriptome sequencing and bioinformatic analyses were performed according to Li [31]. Samples from CK, MI, ET, and ME fruit on day 6 were used for transcriptome sequencing, with three biological replicates per treatment. Total RNA was extracted using the TRIzol method. RNA quality and integrity were assessed before library construction. Qualified libraries were sequenced on the Illumina NovaSeq platform using a paired-end 150 bp strategy. Raw reads were filtered to obtain clean reads, which were mapped to the *Fragaria* × *ananassa* ‘Camarosa’ Genome v1.0.a2 reference genome from the Genome Database for Rosaceae. Differentially expressed genes were identified using DESeq2 with thresholds of |log_2_FoldChange| > 1 and adjusted *p* < 0.05. Comparisons included CK vs. MI, CK vs. ET, CK vs. ME, MI vs. ME, and ET vs. ME. Gene Ontology (GO) and KEGG enrichment analyses were performed.

### 2.7. Reverse Transcription Quantitative PCR (RT-qPCR) Analysis

Ethylene pathway-related genes, including S-adenosylmethionine synthetase (*SAMS*), ACC synthase (*ACS*), ACC oxidase (*ACO*), ethylene response sensor 1 (*ERS1*), and ethylene-insensitive 3 (*EIN3*), were selected for RT-qPCR validation. Primer sequences are listed in Supplementary Table S1. Total RNA was extracted from 150 mg frozen fruit tissue using the Easy Plant RNA Extraction Kit (DR0407050, Zhejiang Easy-Do Biotech Co., Ltd., Hangzhou, China). Reverse transcription was performed using AdvanceFast One-Step RT-gDNA Digestion SuperMix for RT-qPCR (11151ES10, Shanghai Yeasen Biotech Co., Ltd., Shanghai, China). RT-qPCR was carried out using RT-qPCR SYBR Green Master Mix Kit (11201ES03, Shanghai Yeasen Biotech Co., Ltd., Shanghai, China) in a 20 μL reaction volume according to Yang [23] with a two-step amplification protocol. Each sample was analyzed with three technical replicates. *FaActin7* was used as the internal reference gene, and relative expression was calculated using the 2^−ΔΔCT^ method.

### 2.8. Statistical Analysis

Data are presented as the mean ± standard deviation (SD). Statistical analyses were performed using SPSS Statistics 26.0. Physiological and quality data were analyzed by two-way analysis of variance (ANOVA) with treatment and time as fixed factors. Differences among treatment means were assessed using Tukey’s multiple comparisons test. Statistical significance was set at *p* < 0.05. Multivariate analyses of the transcriptomic and metabolomic data were performed using R software (version 4.4.0) or the relevant bioinformatics platforms. Figures were generated using Origin 2021, R software, and Microsoft PowerPoint, as appropriate.

## 3. Results

### 3.1. ME Treatment Accelerated Postharvest Quality Deterioration of Strawberry Fruit

During the shelf-life period, strawberry fruit gradually developed a deeper red color, accompanied by treatment-dependent differences in appearance and quality. Compared with CK fruit, all treated fruit developed red coloration more rapidly, while ME fruit exhibited the most pronounced softening and visual deterioration by day 6 (Figure 1A). Two-way ANOVA showed significant treatment effects for all measured quality attributes. Significant treatment and time interactions were detected for total anthocyanin content and titratable acidity, but not for firmness or soluble solids content. Fruit firmness declined progressively during the shelf-life period, with ME fruit showing the lowest firmness at day 6, indicating more pronounced texture loss under ME treatment (Figure 1B). Weight loss increased in all treatments during the shelf-life period. At both days 3 and 6, weight loss was significantly higher in MI and ME fruit than in CK fruit, whereas ET did not differ significantly from the other treatments (Figure S1). Total anthocyanin content was significantly affected by treatment, time, and their interaction. It increased during the shelf-life period and was highest in ME fruit at days 3 and 6 (Figure 1C). At day 6, ME fruit had a significantly higher soluble solids content than CK fruit, while MI and ET showed intermediate values (Figure 1D). Titratable acidity also varied during the shelf-life period, but the differences among the three treated groups were limited (Figure 1E). This pattern suggests that ethylene exposure and mechanical injury may alter sugar-acid balance during the shelf-life period. Together, these results indicate that mechanical injury and ethylene exposure both affected strawberry postharvest quality, while their combined treatment caused the most severe quality deterioration.

### 3.2. ME Treatment Enhanced Ethylene Biosynthesis and Aggravated Oxidative Membrane Damage

Two-way ANOVA showed significant treatment effects for all ethylene biosynthesis, oxidative damage, and antioxidant enzyme parameters. ME fruit generally showed the highest ethylene production rate during the shelf-life period and had significantly higher ACC content than the other treatments at days 3 and 6 (Figure 2A,B). MI and ET also enhanced these parameters compared with CK, but their effects were less pronounced than those observed in ME fruit. Relative electrolyte leakage increased during the shelf-life period and was highest in ME fruit at day 6 (Figure 3A). MDA and H_2_O_2_ accumulated to higher levels in ME fruit, indicating stronger reactive oxygen species (ROS) accumulation and membrane lipid peroxidation (Figure 3B,C). POD and SOD activities were induced in treated fruit, especially at day 3, but declined to different degrees by day 6 (Figure 3D,E). The concurrent increases in H_2_O_2_, MDA, and electrolyte leakage suggest that the induced antioxidant response was insufficient to fully prevent oxidative membrane damage. Together, these results indicate that ME treatment enhanced ethylene biosynthesis and promoted oxidative membrane damage during the shelf-life period.

### 3.3. ME Treatment Induced Extensive Metabolic Remodeling in Strawberry Fruit

Pairwise orthogonal partial least squares-discriminant analysis (OPLS-DA) analysis showed clear separation between treatment groups on day 6 of the shelf-life period, including CK vs. MI, CK vs. ET, CK vs. ME, MI vs. ME, and ET vs. ME (Figure 4A), indicating that mechanical injury, ethylene exposure, and their combined treatment were associated with distinct metabolomic profiles. In particular, ME samples were separated from both MI and ET samples, suggesting that the ME treatment produced a metabolic profile distinguishable from either single treatment. The number of differentially accumulated metabolites varied considerably among comparisons (Figure 4B). CK vs. ET contained the largest number of differential metabolites, whereas MI vs. ME contained the fewest. This result suggests that the metabolic response associated with ME treatment was not necessarily reflected by a greater number of differential metabolites, but rather by a distinct pattern of metabolite accumulation. Venn analyses further showed that each comparison contained specific metabolites (Figure 4C). Heatmap clustering demonstrated treatment-dependent metabolite accumulation patterns among CK, MI, ET, and ME fruit (Figure 4D). KEGG classification indicated that the differential metabolites were mainly associated with carbohydrate metabolism, amino acid metabolism, chemical structure transformation maps, and the biosynthesis of other secondary metabolites (Figure 4E). Categories related to signal transduction and environmental adaptation were also represented in several comparisons. Overall, these results indicate that ME treatment altered the metabolomic profile of strawberry fruit in a treatment-specific manner, involving carbon metabolism, lipid-related processes, secondary metabolism, and stress-associated pathways.

### 3.4. ME Treatment Induced Broad Transcriptional Responses in Strawberry Fruit

Transcriptome analysis revealed extensive treatment-dependent changes in gene expression on day 6 of the shelf-life period (Figure 5A). Compared with CK, all treatments induced large numbers of differentially expressed genes (DEGs), although the magnitude and direction of transcriptional changes varied among comparisons. In CK vs. MI, 1254 genes were upregulated and 2134 genes were downregulated, whereas CK vs. ET contained 565 upregulated and 4723 downregulated genes. In CK vs. ME, 2078 genes were upregulated and 1934 genes were downregulated. Comparisons between ME and the single-treatment groups also identified large numbers of DEGs, including 2033 upregulated and 792 downregulated genes in MI vs. ME, and 6457 upregulated and 585 downregulated genes in ET vs. ME, indicating substantial transcriptional differences among treatments. Notably, ET vs. ME contained substantially more upregulated DEGs than MI vs. ME, indicating that ME treatment generated a transcriptional state distinct from either single treatment, particularly relative to ET fruit. Venn analysis showed both shared and comparison-specific DEGs across the five comparisons (Figure 5B), suggesting that mechanical injury, ethylene treatment, and their combination induced both common and distinct transcriptional responses. GO classification indicated that DEGs were mainly assigned to biological process categories such as cellular process, metabolic process, biological regulation, response to stimulus, and localization (Figure 5C). To further characterize transcriptional differences associated with the ME treatment, KEGG enrichment analysis was performed for DEGs identified in the MI vs. ME and ET vs. ME comparisons (Figure S2). The enriched pathways included metabolic pathways, biosynthesis of secondary metabolites, phenylpropanoid biosynthesis, plant hormone signal transduction, glutathione metabolism, and several lipid- and carbohydrate-related metabolic pathways. These functional categories indicate that the treatments broadly affected basic cellular activity, metabolic regulation, and stress-related responses. The enrichment of genes related to response to stimulus and metabolic process was consistent with the observed changes in oxidative damage, ethylene biosynthesis, and metabolite accumulation. Together, the transcriptomic results suggest that ME treatment induced a broad transcriptional adjustment involving stress response, metabolism, and cellular structural processes in strawberry fruit.

### 3.5. ME Treatment Affected Phenylpropanoid, Anthocyanin, and Sugar-Acid Metabolism

Genes associated with color development and anthocyanin metabolism were examined based on the transcriptomic data. Genes involved in phenylpropanoid and anthocyanin biosynthesis, including *PAL*, *4CL*, *CHS*, *CHI*, *F3′H*, *DFR*, *ANS*, *UFGT*, and *ACT*, showed treatment-dependent expression patterns (Figure 6A). Several anthocyanin-related genes were more strongly expressed in ME fruit, consistent with the higher total anthocyanin content and accelerated red coloration observed under the ME treatment. MYB transcription factors also showed distinct expression patterns among treatments (Figure 6B). Since MYB proteins are major regulators of anthocyanin structural genes, these changes suggest that anthocyanin accumulation under ME treatment may be associated with both structural gene expression and upstream transcriptional regulation.

### 3.6. ME Treatment Enhanced Ethylene-Related Responses and Altered Hormone-Associated Gene Expression

Genes involved in ethylene biosynthesis and signaling were evaluated at the transcriptional level (Figure 7A). RT-qPCR analysis of selected ethylene pathway genes further supported the transcriptome results. Genes involved in ethylene biosynthesis, including *SAMS*, *ACS*, and *ACO*, displayed treatment-dependent expression patterns, with generally stronger responses in ME fruit (Figure 7B,E,F). The expression changes of *ERS1* and *EIN3* suggested that ethylene sensing and downstream signaling were responsive to ME treatment (Figure 7C,D). These results indicate that ME treatment enhanced ethylene-associated responses at both physiological and transcriptional levels. Genes related to ABA and IAA also exhibited treatment-dependent expression patterns (Figure S3). Several ABA-related genes changed markedly in ME fruit, suggesting possible alterations in ABA metabolism or signaling (Figure S3A). IAA-related genes showed patterns distinct from those in CK, MI, and ET fruit, indicating that auxin-associated signaling may also be involved in the response to ME treatment (Figure S3B). Because ABA and IAA contents were not directly measured, these results should be interpreted as transcriptional evidence of hormone-related pathway changes and require further validation by targeted hormone analysis.

## 4. Discussion

### 4.1. Wound Responses, Ethylene-Associated Regulation, and Oxidative Damage Under ME Treatment

ME treatment was associated with coordinated changes in wound responses, ethylene biosynthesis, oxidative membrane damage, and quality deterioration rather than with the simple amplification of a single quality trait. Mechanical injury may initiate this process by disrupting surface barriers, membrane integrity, and cellular compartmentalization while stimulating endogenous ethylene biosynthesis [32]. In wounded harvested tissues, disruption of cellular organization is often accompanied by redox imbalance, membrane damage, and activation of defense-related metabolism. In this wounded background, ethylene exposure may have acted less as an independent ripening trigger and more as a factor associated with the amplification of stress-related deterioration. Recent reviews emphasize that ethylene biosynthetic regulation is closely involved in plant responses to environmental stresses [33]. Similar observations have been reported in mechanically impacted tomato fruit, where physical impact and wound formation during simulated handling stimulated ethylene production and subsequently altered postharvest physiological responses, supporting the connection between physical impact, ethylene generation, and quality change [34]. In the present study, the coordinated changes in ethylene production, ACC content and ethylene pathway-related genes suggest that ME treatment was associated with strengthened ethylene biosynthesis and ethylene-related signaling. This interpretation is particularly relevant for strawberry because ripening in non-climacteric fruit is not primarily driven by ethylene, unlike that in climacteric fruit. Therefore, the ethylene response observed under ME treatment should not be interpreted as a climacteric-like ripening mechanism. Instead, mechanical injury may have created a stress-sensitive physiological context in which ethylene became more closely connected with deterioration.

Oxidative membrane damage may represent the key physiological bridge between the wound ethylene response and visible quality deterioration. ROS accumulation in harvested fruit is not merely a damage marker; it is closely linked to membrane lipid peroxidation, electrolyte leakage, antioxidant metabolism, and stress signaling [35,36]. In wounded apple fruit, regulation of ROS metabolism was directly associated with maintenance of membrane integrity and wound-related defense responses during storage [37]. In the present study, the concurrent increases in H_2_O_2_, MDA, and relative electrolyte leakage, together with the induction of POD and SOD activities, suggest that antioxidant defenses were activated but were insufficient to prevent oxidative damage. Once membrane integrity is impaired, water loss, solute leakage, metabolic disorder, and enzyme–substrate contact may be intensified, providing a plausible explanation for the transition from local injury to whole-fruit deterioration [38].

Such oxidative imbalance may further contribute to structural weakening during the shelf-life period [39]. Strawberry softening is mainly driven by changes in cell wall structure and composition, including pectin modification, hemicellulose remodeling, and reduced cell-to-cell adhesion [40]. In the present study, ME fruit showed the most pronounced firmness loss and visual deterioration, suggesting that structural weakening was accelerated under the combined treatment. Ethylene perception may also be connected with this process, as 1-MCP treatment has been reported to delay softening, preserve ionically bound pectin and hemicellulose fractions, and affect key cell wall metabolism enzymes and genes in harvested strawberry fruit [41]. However, because cell wall-degrading enzyme activities, cell wall polysaccharide composition, and cell wall-related gene expression were not directly analyzed in the present study, cell wall remodeling should be considered a possible downstream process rather than a directly demonstrated mechanism.

Taken together, the results suggest that mechanical injury and exogenous ethylene jointly strengthened ethylene-associated responses in strawberry fruit. Under this condition, wound responses, ethylene biosynthesis, ROS accumulation, and membrane damage became closely interconnected, forming an upstream physiological framework associated with accelerated postharvest deterioration. These associations require further validation using ethylene inhibitors, ROS modulators, targeted cell wall analyses, and hormone quantification. Nevertheless, they provide a physiological context for interpreting the metabolic and transcriptional changes discussed below.

### 4.2. Metabolic and Transcriptional Reprogramming Associated with the ME Treatment

The multi-omics results suggest that the strengthened ethylene-associated responses under ME treatment were accompanied by distinct metabolic and transcriptional reprogramming rather than a simple intensification of all postharvest changes. In the metabolomic analysis, ME samples were clearly separated from MI and ET samples in pairwise OPLS-DA, indicating a distinct metabolic profile. However, CK vs. ME did not contain the largest number of differentially accumulated metabolites under the applied screening criteria. Therefore, the metabolic impact of ME treatment is better interpreted as a treatment-specific change in selected metabolic pathways rather than a general increase in the number of altered metabolites.

Phenylpropanoid and anthocyanin metabolism represented one of the major secondary metabolic branches associated with this response. In ME fruit, increased total anthocyanins were consistent with changes in genes involved in phenylpropanoid and anthocyanin biosynthesis, including *PAL*, *4CL*, *CHS*, *CHI*, *F3′H*, *DFR*, and *ANS* [42]. MYB transcription factors also showed treatment-dependent expression patterns, suggesting that upstream transcriptional regulation may contribute to the observed anthocyanin-related changes [43]. Since anthocyanins are the main pigments responsible for strawberry red coloration, these results provide a molecular explanation for the enhanced red color observed under ME treatment. At the same time, phenylpropanoid metabolism is also involved in antioxidant defense and stress adaptation, meaning that the accumulation of anthocyanins may reflect both pigmentation and a stress-related secondary metabolic response [44,45]. Because ethylene treatment alone also promoted anthocyanin accumulation and coloration-related changes, the enhanced anthocyanin metabolism observed under ME treatment may be associated with strengthened ethylene responsiveness in addition to stress adaptation.

Sugar-acid metabolism represented another important branch of the ME response. Strawberry flavor is strongly influenced by the balance between soluble sugars and organic acids, while carbon metabolism also supplies substrates for energy production and secondary metabolism [46,47]. The altered expression of *SWEET*, *SUT*, *CWIN*, *SUS*, *HK*, *PGM*, *PK*, *PEPC*, *CS*, and *MDH* suggests that sucrose transport, sugar cleavage, glycolysis, and organic acid metabolism were affected [48]. These transcriptional changes were consistent with the observed changes in soluble solids content and titratable acidity. However, because individual sugars and organic acids were not quantified, these results should be interpreted as transcriptional evidence of altered sugar-acid-related pathways rather than direct proof of specific compositional shifts.

The metabolomic classification also included lipid metabolism-related categories, which may be relevant to the oxidative membrane damage discussed in Section 4.1 [49]. However, lipid metabolism was not further resolved at the level of specific lipid species in the present study. A more cautious interpretation is that lipid-related metabolic changes may be associated with the membrane deterioration phenotype, as suggested by the increases in MDA and relative electrolyte leakage. Future targeted lipidomic analysis would be required to clarify whether specific membrane lipid classes participate in ME-induced oxidative damage.

Hormone-associated transcriptional changes further suggest that ME-induced metabolic reprogramming may involve a broader regulatory network. In strawberry and other non-climacteric fruit, ABA is a major regulator of ripening and quality formation, whereas auxin is generally associated with early fruit development and delayed ripening [16]. In this study, ABA-related genes, including *ZEP*, *NCED*, *ABA2*, *AAO3*, *CYP707A*, *UGT*, *BG1*, and *ABCG*, showed altered expression patterns, suggesting that ABA biosynthesis, catabolism, conjugation, release, or transport may have been affected [50]. Similarly, changes in *TAA*, *YUCCA*, *TIR*, *AUX/IAA*, and *ARF* indicate possible alteration of auxin biosynthesis and signaling [51]. These results suggest that combined mechanical injury and ethylene exposure may intersect with endogenous hormone-related regulatory pathways. Nevertheless, the hormone-related results should also be interpreted cautiously. Because ABA and IAA contents were not directly measured, these data provide transcriptional evidence of hormone-related pathway changes rather than direct evidence of hormone accumulation or activity.

Overall, the downstream response to ME treatment can be interpreted as selective metabolic and transcriptional reprogramming associated with strengthened ethylene-associated responses. Phenylpropanoid and anthocyanin pathways, sugar-acid metabolism, hormone-related transcriptional regulation, and redox-related processes were all affected to varying degrees. These findings suggest that enhanced ethylene responsiveness may serve as an important link connecting wound-induced stress with broader metabolic remodeling during postharvest strawberry deterioration. The experiment was conducted at 20 °C to accelerate fruit deterioration and obtain measurable treatment differences within 6 days, whereas strawberries are normally stored and transported under cold conditions. Microbial load was not measured and may have contributed to the deterioration of injured fruit. Future studies should compare room temperature with cold storage and monitor microbial changes.

## 5. Conclusions

Combined mechanical injury and ethylene exposure caused the most pronounced quality deterioration in strawberry fruit, accompanied by increased ethylene production, ACC accumulation, ROS accumulation, membrane lipid peroxidation, and membrane permeability. Day-6 transcriptomic and metabolomic analyses further revealed treatment-associated changes in phenylpropanoid and anthocyanin metabolism, sugar-acid-related pathways, and hormone-associated gene expression. These findings support an association between wound responses, ethylene-related processes, oxidative membrane damage, and metabolic remodeling during strawberry deterioration. However, because early molecular responses, hormone contents, and cell wall biochemical changes were not directly determined, the proposed relationships require further validation. From a practical perspective, mechanical damage could be reduced by packing strawberries in a reusable harvest crate with a removable multifunctional liner. The liner could provide cushioning at the bottom and sides of the crate, reduce fruit movement during transport, and contain separate zones for moisture control and ethylene removal. Future studies should compare conventional foam liners with ventilated molded fiber liners and active liners under realistic harvest, stacking, vibration, precooling, and cold storage conditions. Bruising, package gases, condensation, microbial decay, and fruit quality should be evaluated to identify a suitable system for commercially harvested strawberries.

## Figures and Tables

**Figure 1:**
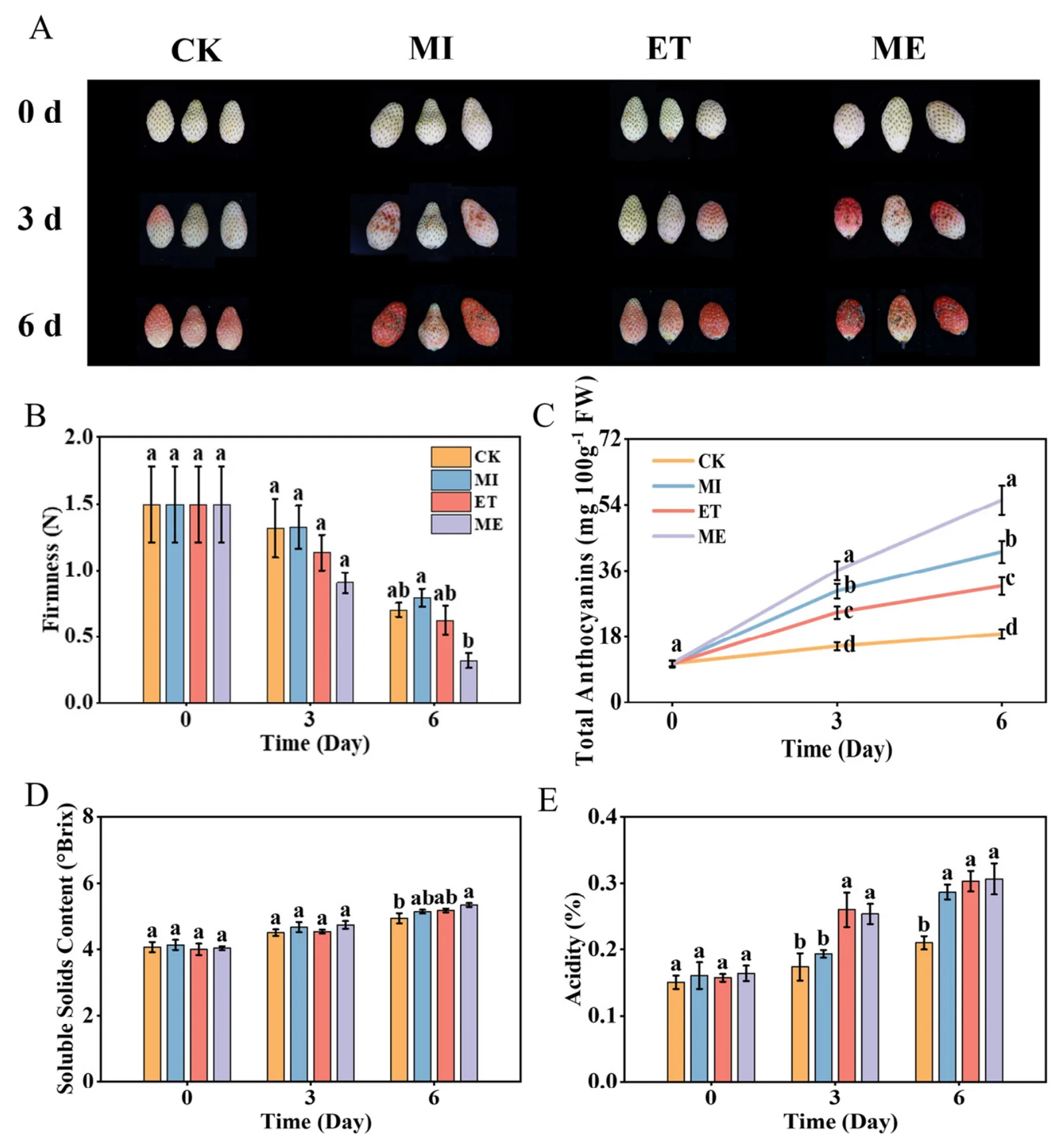
Effects of mechanical injury and ethylene treatment on postharvest quality attributes of strawberry fruit. (**A**) Representative appearance of strawberry fruit during the shelf-life period. (**B**) Firmness. (**C**) Total anthocyanin content. (**D**) Soluble solids content. (**E**) Titratable acidity. CK, control; MI, mechanical injury; ET, ethylene treatment; ME, mechanical injury combined with ethylene treatment. Values are presented as means ± SD of three independent biological replicates (*n* = 3). Different letters indicate significant differences among treatments at the same time according to Tukey-adjusted simple-effect comparisons (*p* < 0.05).

**Figure 2:**
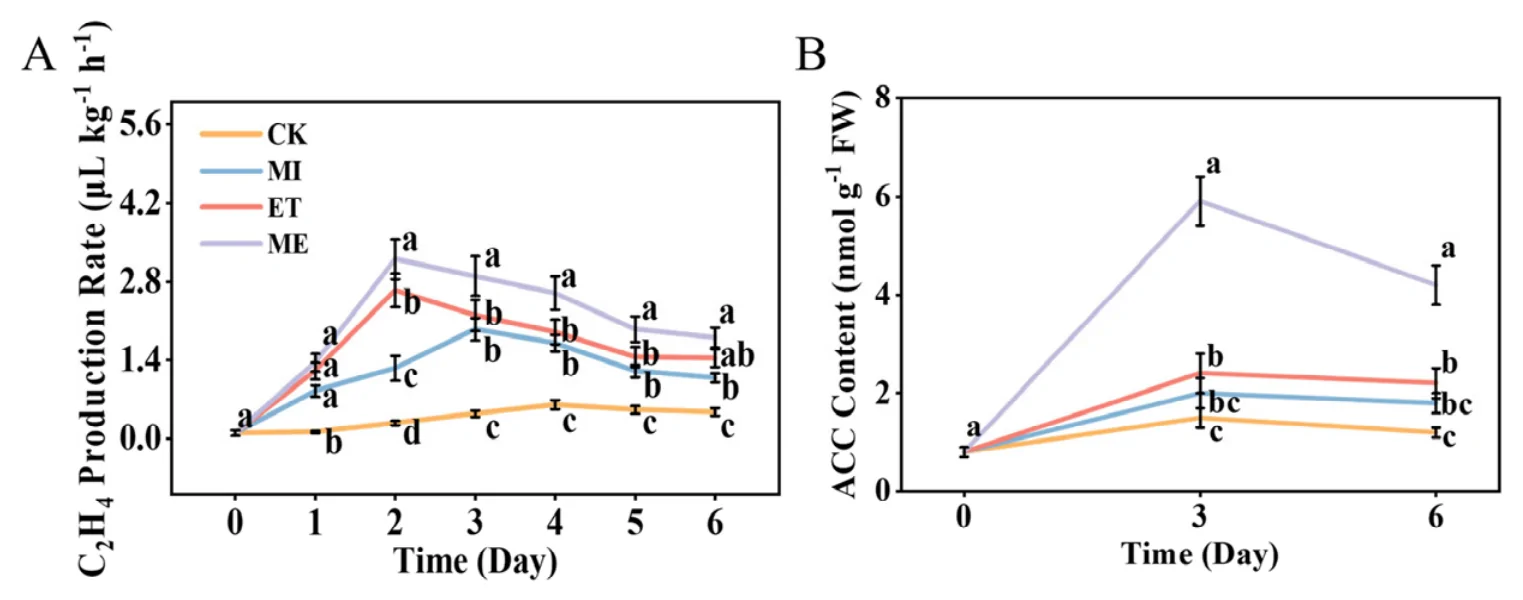
Effects of mechanical injury and ethylene treatment on ethylene biosynthesis-related parameters in strawberry fruit. (**A**) Ethylene production rate. (**B**) ACC content. Different letters indicate significant differences among treatments at the same time at *p* < 0.05, *n* = 3.

**Figure 3:**
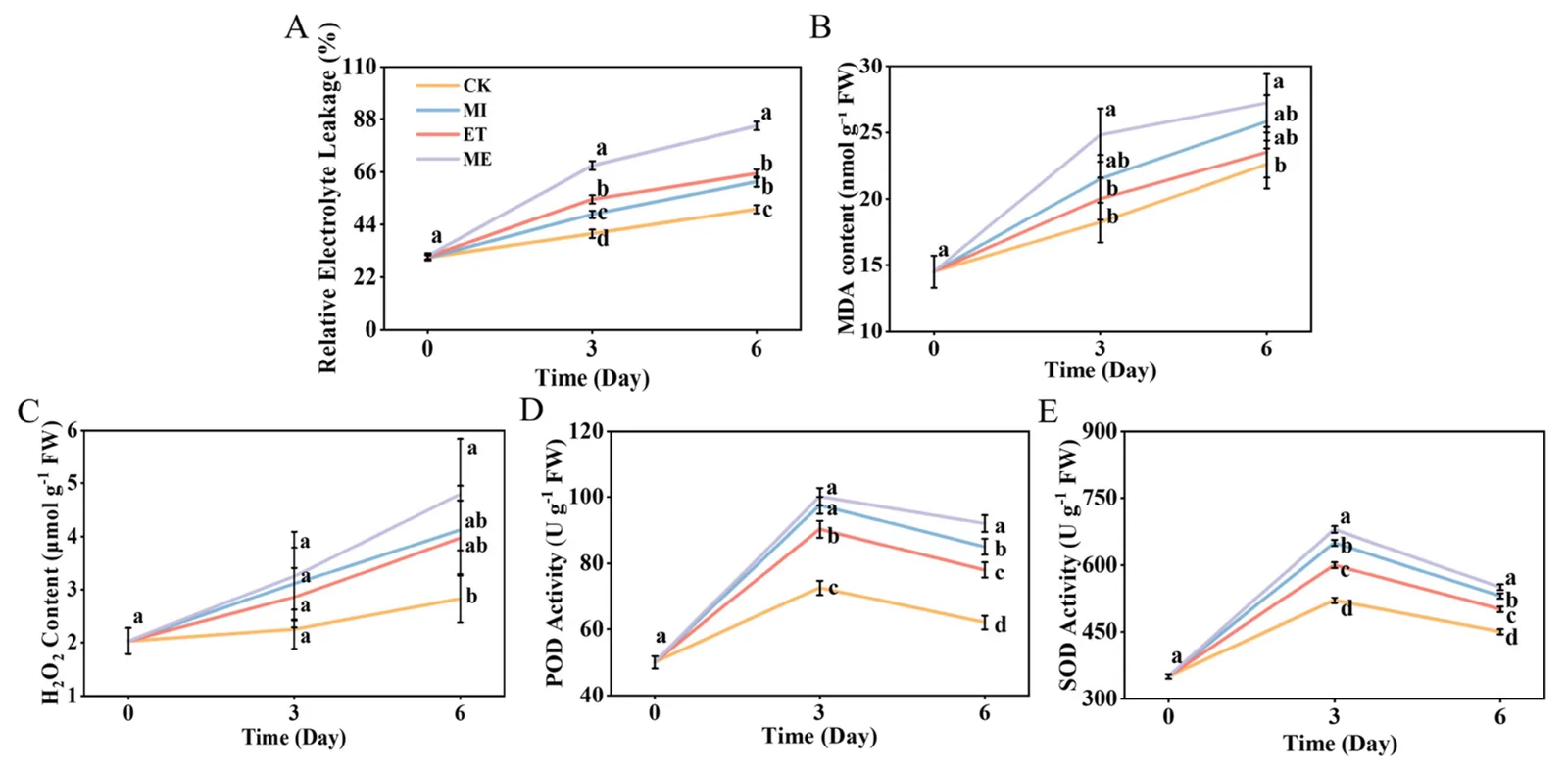
Effects of mechanical injury and ethylene treatment on oxidative damage and antioxidant enzyme activities in strawberry fruit. (**A**) Relative electrolyte leakage. (**B**) MDA content. (**C**) H_2_O_2_ content. (**D**) POD activity. (**E**) SOD activity. Different letters indicate significant differences among treatments at the same time at *p* < 0.05, *n* = 3.

**Figure 4:**
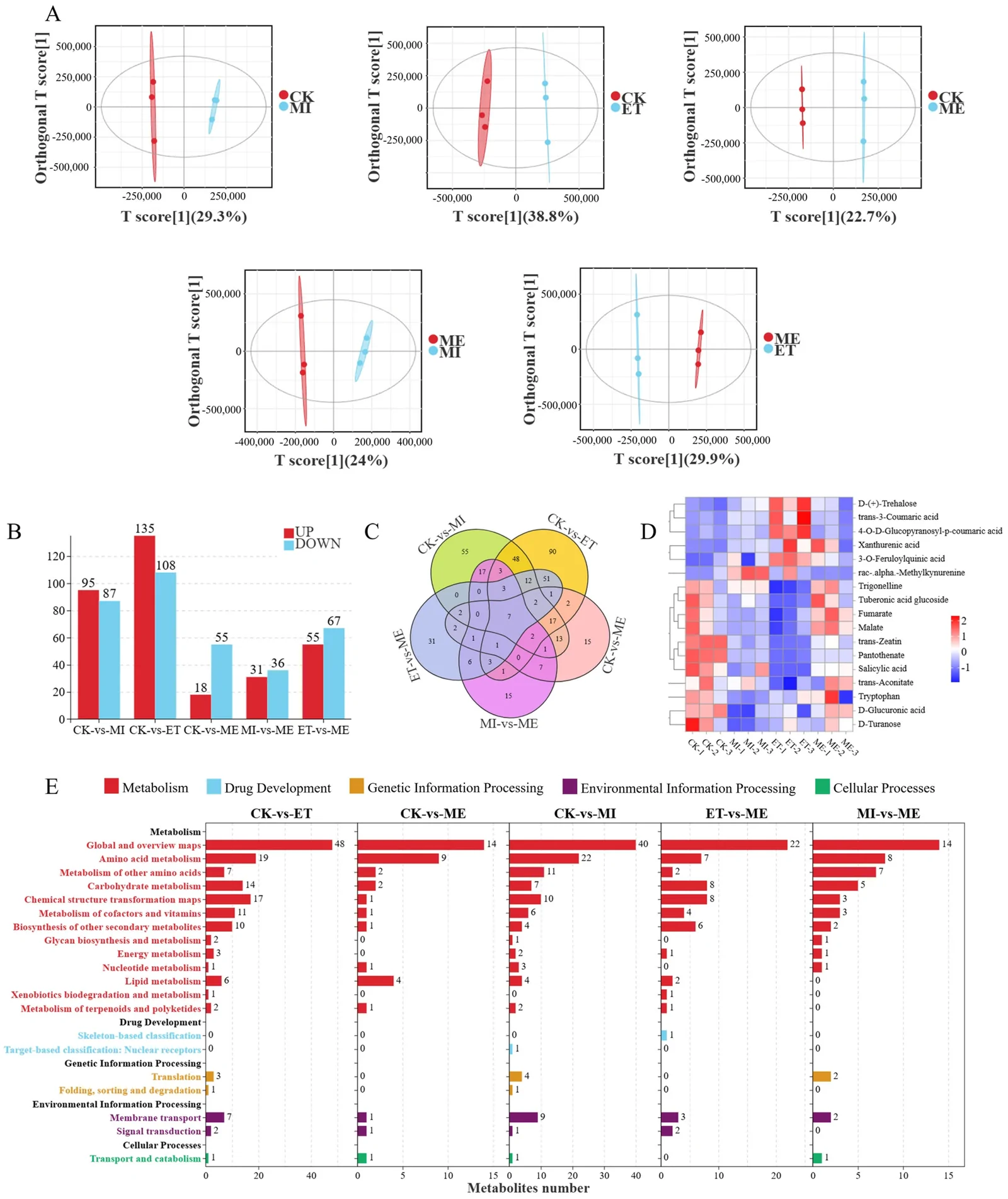
Metabolomic profiling of strawberry fruit under mechanical injury and ethylene treatments. (**A**) OPLS-DA score plot of metabolomic profiles among CK, MI, ET, and ME groups. (**B**) Number of differentially accumulated metabolites in different comparison groups. (**C**) Venn analysis of differentially accumulated metabolites. (**D**) Heatmap clustering of representative differentially accumulated metabolites. (**E**) KEGG classification of differentially accumulated metabolites. Heatmaps were generated based on log_2_FoldChange values. Blue and red indicate decreased and increased relative abundance.

**Figure 5:**
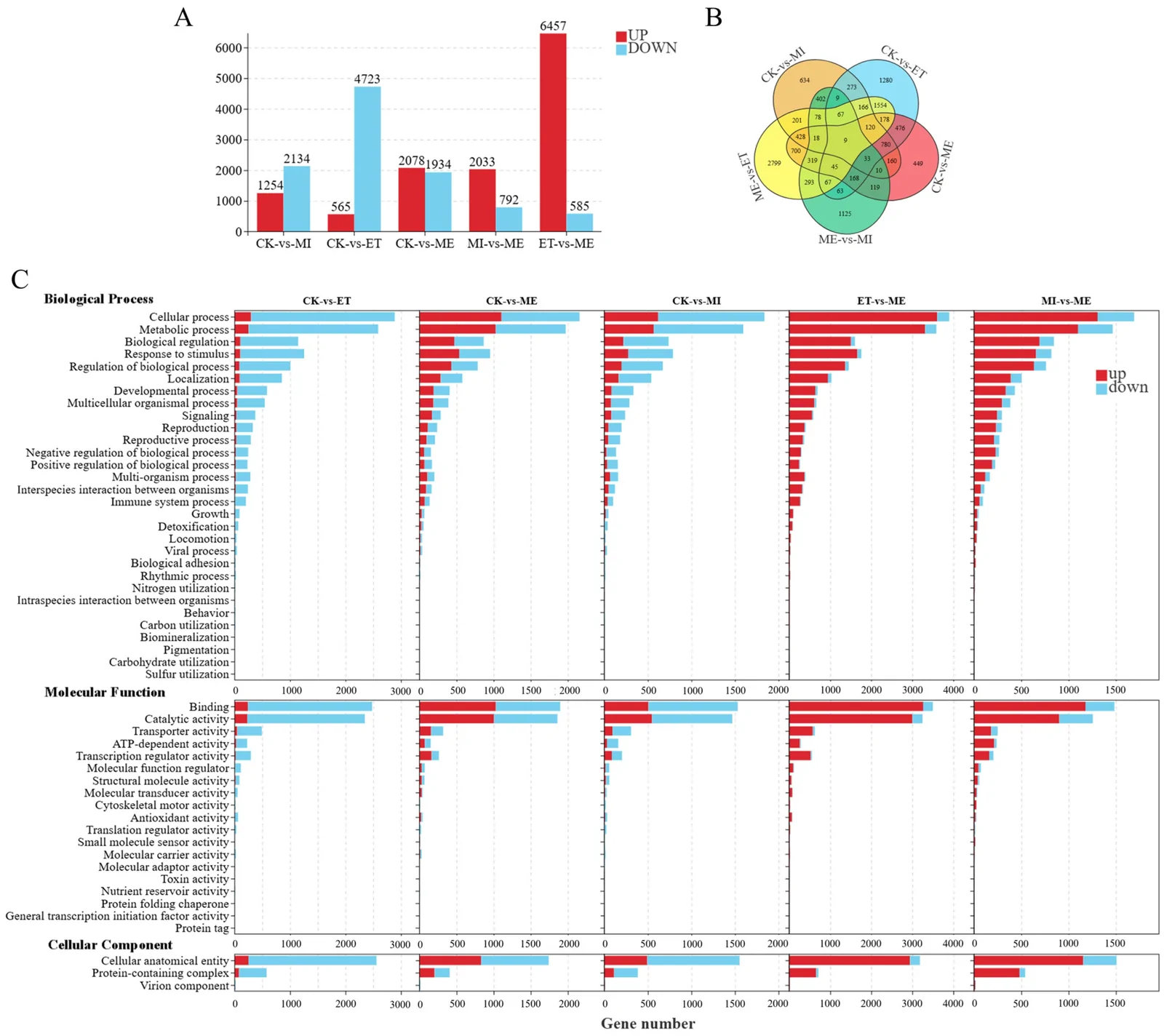
Transcriptomic analysis of strawberry fruit under mechanical injury and ethylene treatments. (**A**) Number of upregulated and downregulated differentially expressed genes in different comparison groups. (**B**) Venn analysis of differentially expressed genes among comparisons. (**C**) GO classification of differentially expressed genes. Differentially expressed genes were identified using |log_2_FoldChange| > 1 and adjusted *p* < 0.05.

**Figure 6:**
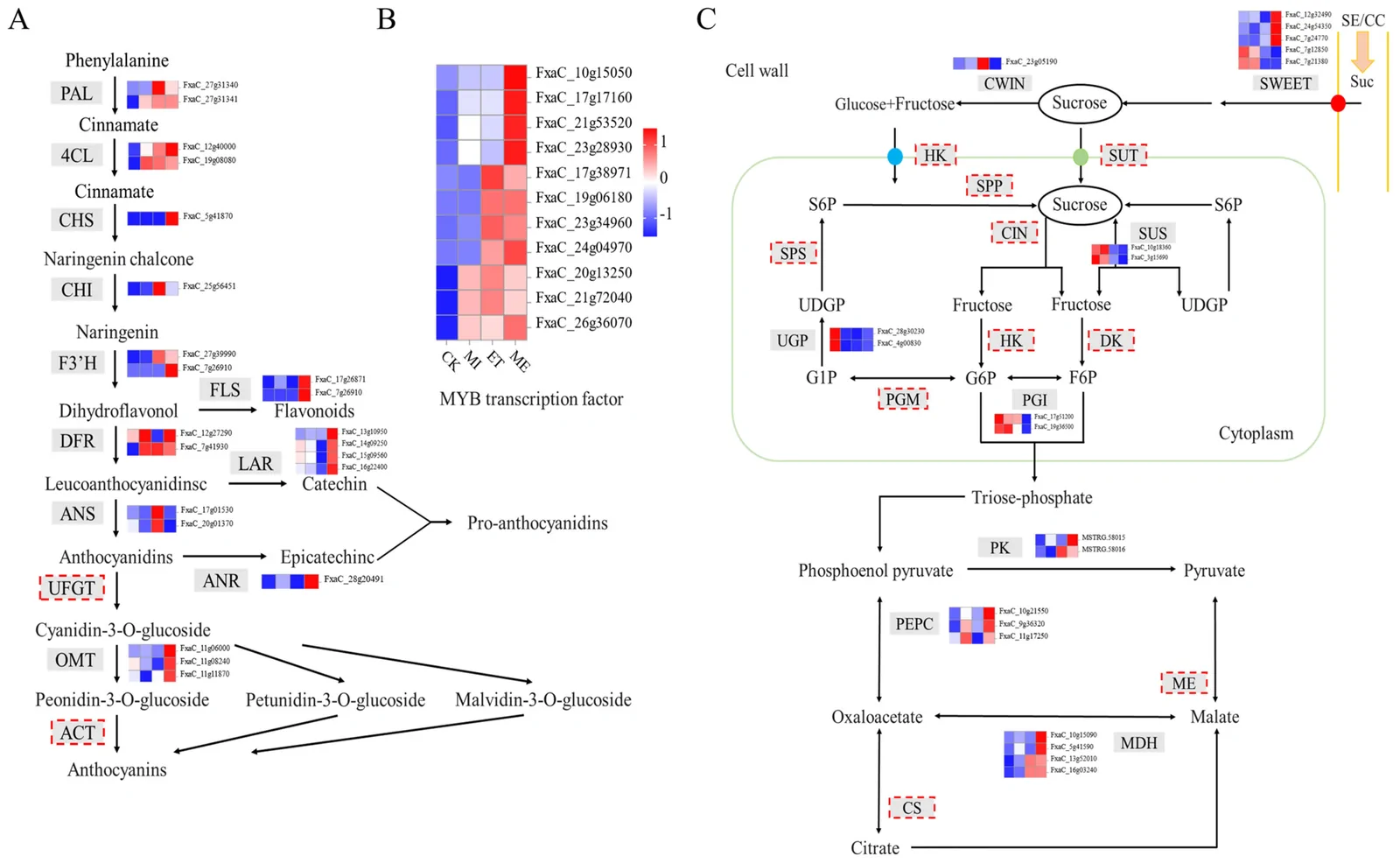
Regulation of phenylpropanoid, anthocyanin, and sugar-acid metabolism pathways under mechanical injury and ethylene treatments. (**A**) Expression changes of genes involved in phenylpropanoid and anthocyanin biosynthesis pathways. (**B**) Heatmap of MYB transcription factors associated with anthocyanin regulation. (**C**) Expression changes of genes involved in sucrose metabolism, glycolysis, and organic acid metabolism. CK, control; MI, mechanical injury; ET, ethylene treatment; ME, mechanical injury combined with ethylene treatment. Gene expression levels are shown as normalized transcript abundance. Heatmaps were generated based on log_2_FoldChange values. Blue and red indicate decreased and increased relative expression. The red dashed line represents |log_2_FoldChange| = 0. The full names and functional annotations of the pathway-related genes are provided in Supplementary Table S2.

**Figure 7:**
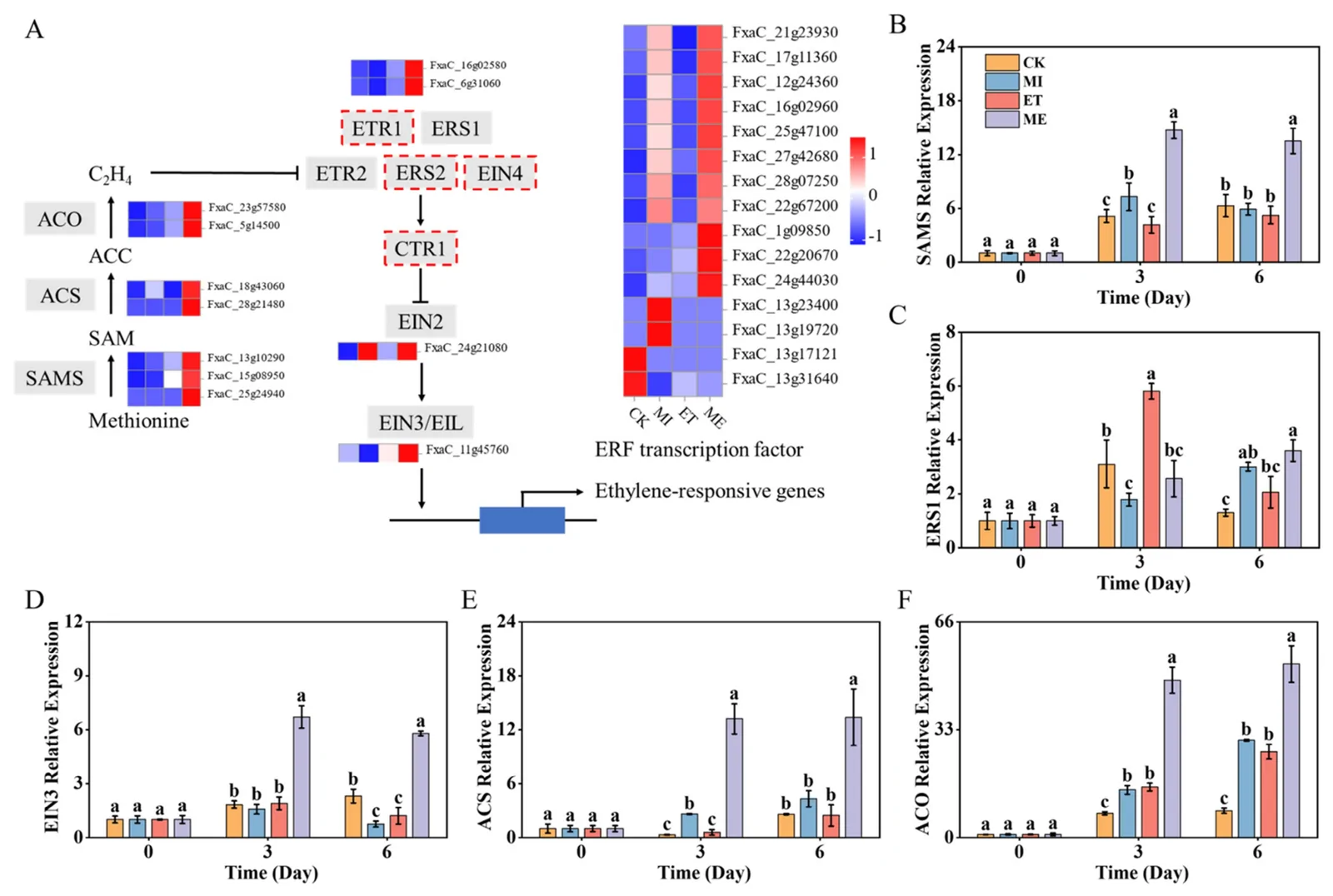
Regulation of ethylene biosynthesis, ethylene signaling, and hormone-related gene expression under mechanical injury and ethylene treatments. (**A**) Expression changes of genes involved in ethylene biosynthesis. (**B**) SAMS, (**C**) ERS1, (**D**) EIN3, (**E**) ACS, and (**F**) ACO. FaActin7 was used as the internal reference gene. Heatmaps were generated based on log_2_FoldChange values. Blue and red indicate decreased and increased relative expression. The red dashed line represents |log_2_FoldChange| = 0. Different letters indicate significant differences among treatments at the same time at *p* < 0.05, *n* = 3.

## Data Availability

The high-throughput omic data generated in this study have been deposited in the NCBI Sequence Read Archive under accession number PRJNA1479201. Supplementary Table S2 is provided in the Supplementary Materials. Further data are available from the corresponding author upon reasonable request.

## Supplementary Materials

The following supporting information can be downloaded at: https://www.mdpi.com/article/10.3390/foods15193380/s1, Figure S1: Changes in fruit weight percentage across different groups. CK, control; MI, mechanical injury; ET, ethylene treatment; ME, mechanical injury combined with ethylene treatment. Different letters indicate significant differences among treatments at the same time at *p* < 0.05, *n* = 3; Figure S2: KEGG enrichment analysis of upregulated and downregulated DEGs in ME fruit compared with the single treatment groups. (A) Enriched pathways of upregulated DEGs in ME fruit relative to MI and ET fruit. (B) Enriched pathways of downregulated DEGs in ME fruit relative to MI and ET fruit. The *x*-axis represents the gene ratio, bubble size indicates the number of DEGs enriched in each pathway, and bubble color represents the significance level [−log_10_(Q value)]; Figure S3: Expression changes of ABA- and IAA-related pathway genes under mechanical injury and ethylene treatments. (A) Expression changes of genes involved in ABA biosynthesis, metabolism, transport, and signaling. (B) Expression changes of genes involved in IAA biosynthesis and signaling. Normalized transcript abundance is shown for each treatment group; Table S1: Primer sequences used for RT-qPCR analysis; Table S2: Full names and functional categories of genes shown in Figure 6, Figure 7 and Figure S3.
