# Supplementary material for: Mechanical Injury Enhances Ethylene-Associated Transcriptional and Metabolic Responses of Strawberry Fruit During Shelf-Life Period
Source: Foods. 2026 Sep 23;15(19):3380. doi: 10.3390/foods15193380 (PMC13664320; doi:10.3390/foods15193380)
Supplement: Supplementary file 1 [file foods-15-03380-s001.zip › Supplementary Materials.pdf]

Supplementary Materials:

Table S1. Primer sequences used for RT-qPCR analysis.

Gene ID	Primer name	Sequence (5′–3′)
FxaC_23g57580	ACO-qpcr-F	CTTGGTGACCAGCTTGAGGT
	ACO-qpcr-R	CAAAGATGGTGCCGGGTAGA
FxaC_18g43060	ACS-qpcr-F	TGGAAGCAAGGCACACAAAAT
	ACS-qpcr-R	GCAAAGCAAACCCTGAACCA
FxaC_13g10290	SAMS-qpcr-F	CTGCTGAACCAGCGCACTTA
	SAMS-qpcr-R	GTTGAAATCGCCGCTCCTTC
FxaC_16g02580	ERS1-qpcr-F	AGAACTCATCTGATAGTGGTGATG
	ERS1-qpcr-R	ACAAGTGCCATGCTTTGAGG
FxaC_11g45760	EIN3-qpcr-F	CCGAGAAGAAGCCCTCATCC
	EIN3-qpcr-R	TGGCTCTACATCTATCCTCCTGT
FaActin7	FaActin7-F	GCCCTTGACTACGAACAAGAA
	FaActin7-R	CCTGAATCTCTCAGCTCCAATC

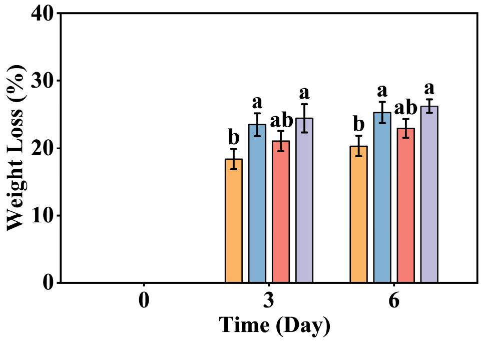

Figure S1. Changes in fruit weight percentage across different groups. CK, control; MI, mechanical injury; ET, ethylene treatment; ME, mechanical injury combined with ethylene treatment. Different letters indicate significant differences among treatments at the same time at $p < 0.05$, $n = 3$.

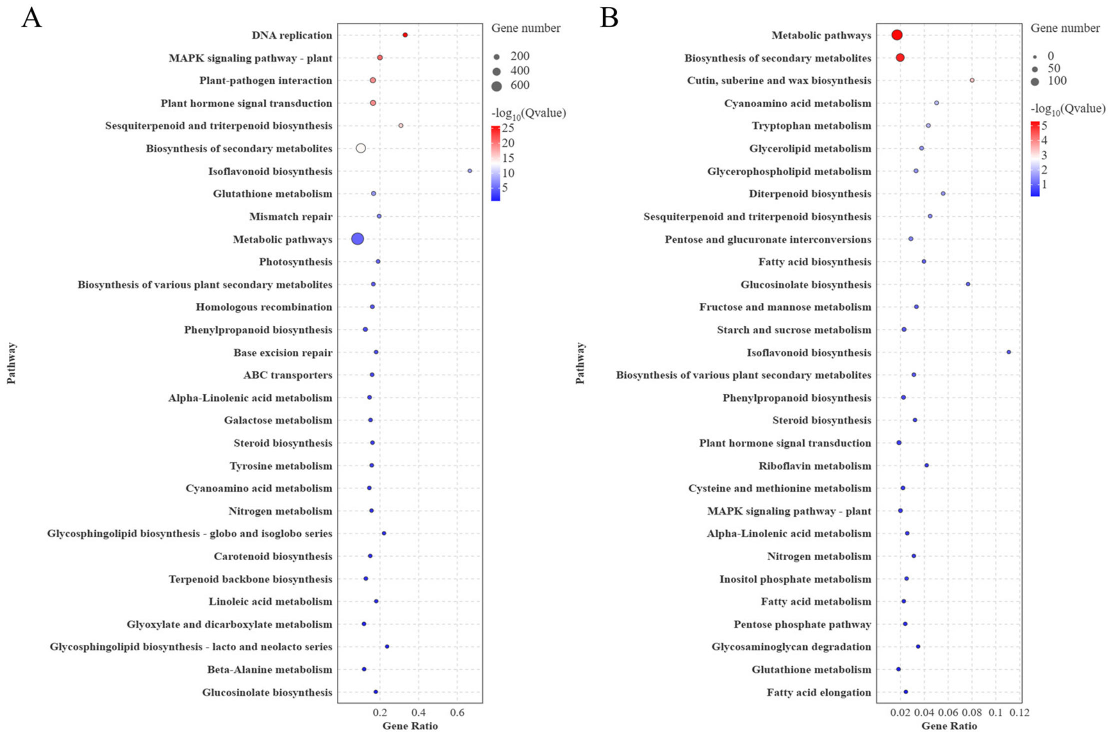

Figure S2. KEGG enrichment analysis of upregulated and downregulated DEGs in ME fruit compared with the single treatment groups.

(A) Enriched pathways of upregulated DEGs in ME fruit relative to MI and ET fruit. (B) Enriched pathways of downregulated DEGs in ME fruit relative to MI and ET fruit. The *x*-axis represents the gene ratio, bubble size indicates the number of DEGs enriched in each pathway, and bubble color represents the significance level [$-\log_{10}$(Q value)].

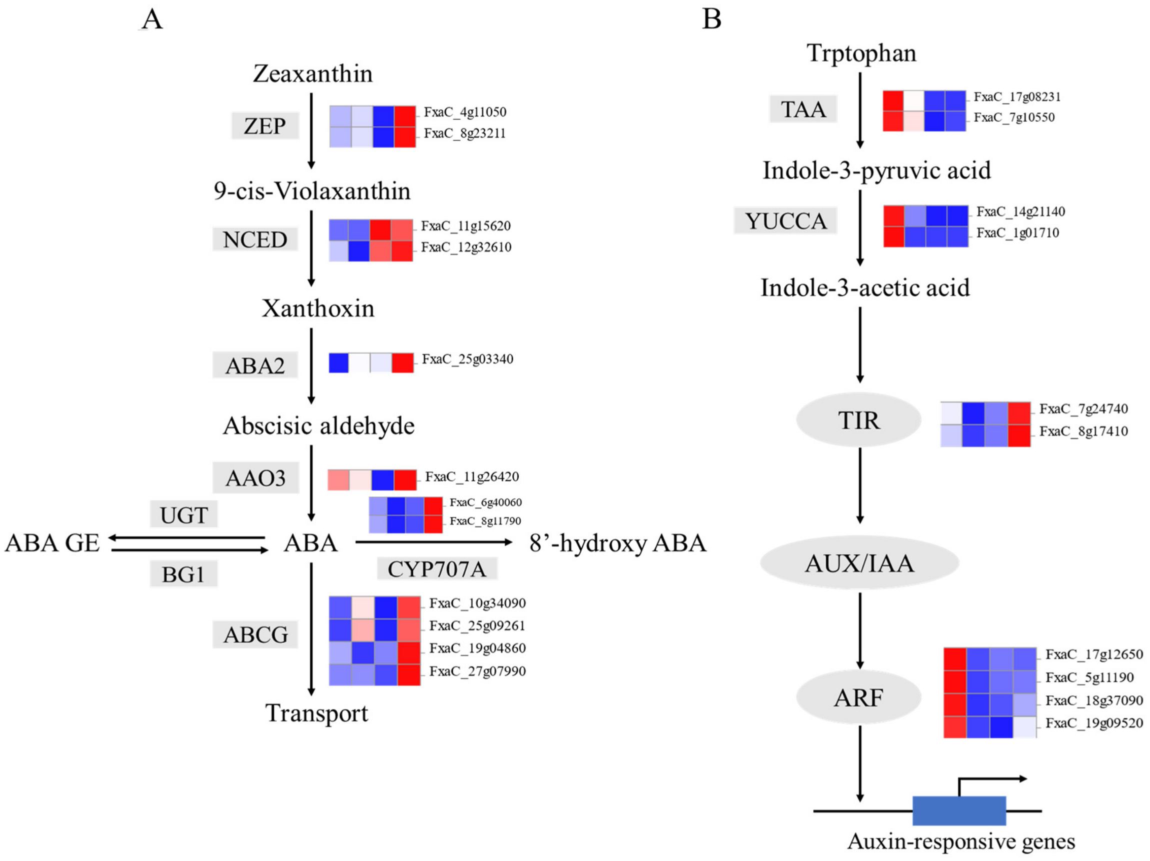

Figure S3. Expression changes of ABA- and IAA-related pathway genes under mechanical injury and ethylene treatments.
(A) Expression changes of genes involved in ABA biosynthesis, metabolism, transport, and signaling. (B) Expression changes of genes involved in IAA biosynthesis and signaling. Normalized transcript abundance is shown for each treatment group.
